# Influence of stimulation frequency on brain-derived neurotrophic factor and cathepsin-B production in healthy young adults

**DOI:** 10.1007/s00360-024-01566-0

**Published:** 2024-05-31

**Authors:** Yuichi Nishikawa, Hiroyuki Sakaguchi, Tatsuya Takada, Noriaki Maeda, Allison Hyngstrom

**Affiliations:** 1 Faculty of Frontier Engineering, Institute of Science & Engineering, Kanazawa University, Kakuma-machi, Kanazawa, Kanazawa, 920-1192 Japan; 2 Graduate School of Frontier Engineering, Kanazawa University, Kanazawa, Japan; 3 MTG Co., Ltd, Nagoya, Japan; 4 Department of Sport Rehabilitation, Graduate School of Biomedical and Health Sciences, Hiroshima University, Hiroshima, Japan; 5 Department of Physical Therapy, Marquette University, Milwaukee, WI USA

**Keywords:** Myokine, Electrical muscle stimulation, Brain-derived neurotrophic factor, cathepsin-B

## Abstract

Electrical muscle stimulation (EMS) has been shown to stimulate the production of myokines (i.e., brain-derived neurotrophic factor (BDNF)), but the most effective EMS parameters for myokine production have not been fully elucidated. The purpose of this study was to quantify the optimal EMS frequency for stimulating myokine production. This study included sixteen young adults (male, *n* = 13, age = 27.3 ± 5.5 years). Participants underwent four EMS interventions (20 min each) with the following conditions: (1) 4 Hz, (2) 20 Hz, (3) 80 Hz, and (4) control (no intervention). Blood samples were obtained before and immediately after EMS. For the control condition, blood samples were taken before and after 20 min of quiet sitting. BDNF and cathepsin-B levels were analyzed in serum. Compared to preintervention levels, stimulation at 20 Hz resulted in significantly greater postintervention cathepsin-B and BDNF levels (*p* < 0.01). On the other hand, the control condition did not result in a significant change between pre- and posttreatment. Furthermore, stimulation at 20 Hz caused significantly larger increases in cathepsin-B and BDNF levels than stimulation at 4–80 Hz or the control condition (*p* < 0.05). In conclusion, stimulation at 20 Hz effectively causes a robust cathepsin-B and BDNF response. Based on these results, we suggest a new strategy for rehabilitation of people with neurological disorders.

## Introduction

Electrical muscle stimulation (EMS) has recently been reported to have the potential to improve neuromuscular and cardiopulmonary function not only in healthy adults but also in elderly people and patients with several diseases (Banerjee 2010; Caulfield et al. 2013; Miyamoto et al. 2016), and it is beginning to be considered an adjunct therapy or alternative to exercise that requires voluntary effort. EMS induces muscle contraction by sending electrical pulses to the target muscle via surface electrodes and thus does not require voluntary effort for muscle contraction. Therefore, EMS may be an effective means of providing the benefits of neuromuscular activation to low-functioning individuals. While several previous studies have focused on the effects of EMS on muscle strength (Bélanger et al. 2000; Maffiuletti et al. 2011; Jones et al. 2016), recent years have seen a focus on its benefits on metabolic (Miyamoto et al. 2015, 2018a) and neurological functions (Nishikawa et al. 2019, 2021c, 2023), by which EMS could further enhance neuromuscular activation in a variety of patient populations.

Myokines have been suggested to underlie the effects of EMS on neuromuscular function. Myokines are physiologically active substances secreted by various types of skeletal muscles during muscle contraction, and more than 300 types of myokines have been identified (Hartwig et al. 2014). Brain-derived neurotrophic factor (BDNF) is a myokine that has been widely studied. There is a large body of evidence examining BDNF expressed in the hippocampus, and it has been implicated in learning, memory, and cognition (Tyler et al. 2002). Interestingly, BDNF expression has been found not only in the brain but also in skeletal muscle cells (Matsumoto et al. 2021), indicating a potential link between exercise and cognitive function. Cathepsin-B is released from skeletal muscle cells in response to exercise in humans and animals, and it has been recently reported to penetrate the blood‒brain barrier and promote BDNF production in the brain (Moon et al. 2016). In an intervention study in humans, Moon et al. found a positive correlation between increased cathepsin-B levels and improved cognitive function as a result of physical training (Moon et al. 2016). BDNF production has been reported to increase in response to EMS as well as voluntary training (Miyamoto et al. 2018b; Nishikawa et al. 2021b, 2023). However, to the best of our knowledge, there are no reports on cathepsin-B and whether its production is increased by EMS. Furthermore, EMS involves multiple parameters (such as frequency and pulse width), and the most effective parameters for inducing BDNF and cathepsin-B production are unknown. The identification of effective EMS parameters to produce these myokines is crucial for developing alternative interventions for the treatment of dementia and other neurodegenerative disorders. One of the variables influencing the efficacy of EMS is the frequency (Moritani et al. 1985; Doucet et al. 2012). Most clinical regimens utilize frequencies between 20 and 50 Hz (Baker et al. 1988; de Kroon et al. 2005). Moritani et al. reported that at frequencies above 50 Hz, exerted torque decreased after approximately 30 s of stimulation, while 20 Hz was associated with the ability to maintain a high level of exerted torque (Moritani et al. 1985). Mettler et al. also reported that low-frequency EMS (20 Hz) is more favorable for fatigue and muscle torque production than high-frequency EMS (60 Hz) (Mettler et al. 2018). Previously, we found that low-frequency EMS (20 Hz) increased serum BDNF levels and that changes in BDNF expression were dependent on the amount of stimulated muscle (Nishikawa et al. 2021b, 2023). Another study reported that EMS at 4 Hz increased serum BDNF levels (Miyamoto et al. 2018b). However, there have been no reports comparing BDNF and cathepsin-B expression at different frequencies. It is necessary to determine the most effective parameters for inducing BDNF and cathepsin-B production to determine the prescriptive dose of EMS for people with dementia and other disorders.

The purpose of this study was to identify the EMS frequencies that are effective for increasing BDNF and cathepsin-B levels in young adults. We hypothesized that EMS would increase BDNF and cathepsin-B levels compared to the unstimulated condition, and their production would increase in a frequency-dependent manner.

## Materials and methods

### Participants

Sixteen healthy young adults participated in this study (male, *n* = 13, age = 27.3 ± 5.5 years, height = 171.0 ± 10.0 cm, weight = 61.3 ± 9.8 kg). The inclusion criteria were as follows: able to give informed consent and able to walk independently. Individuals with a history of diabetes, neuromuscular diseases, cardiovascular diseases or orthopedic conditions were excluded. The Kanazawa University Committee on Ethics in Research approved all procedures in accordance with the Declaration of Helsinki (approval no. 2020-003 (6130)). Participants gave informed written consent to participate in this study.

### Experimental procedures

**Fig. 1:**
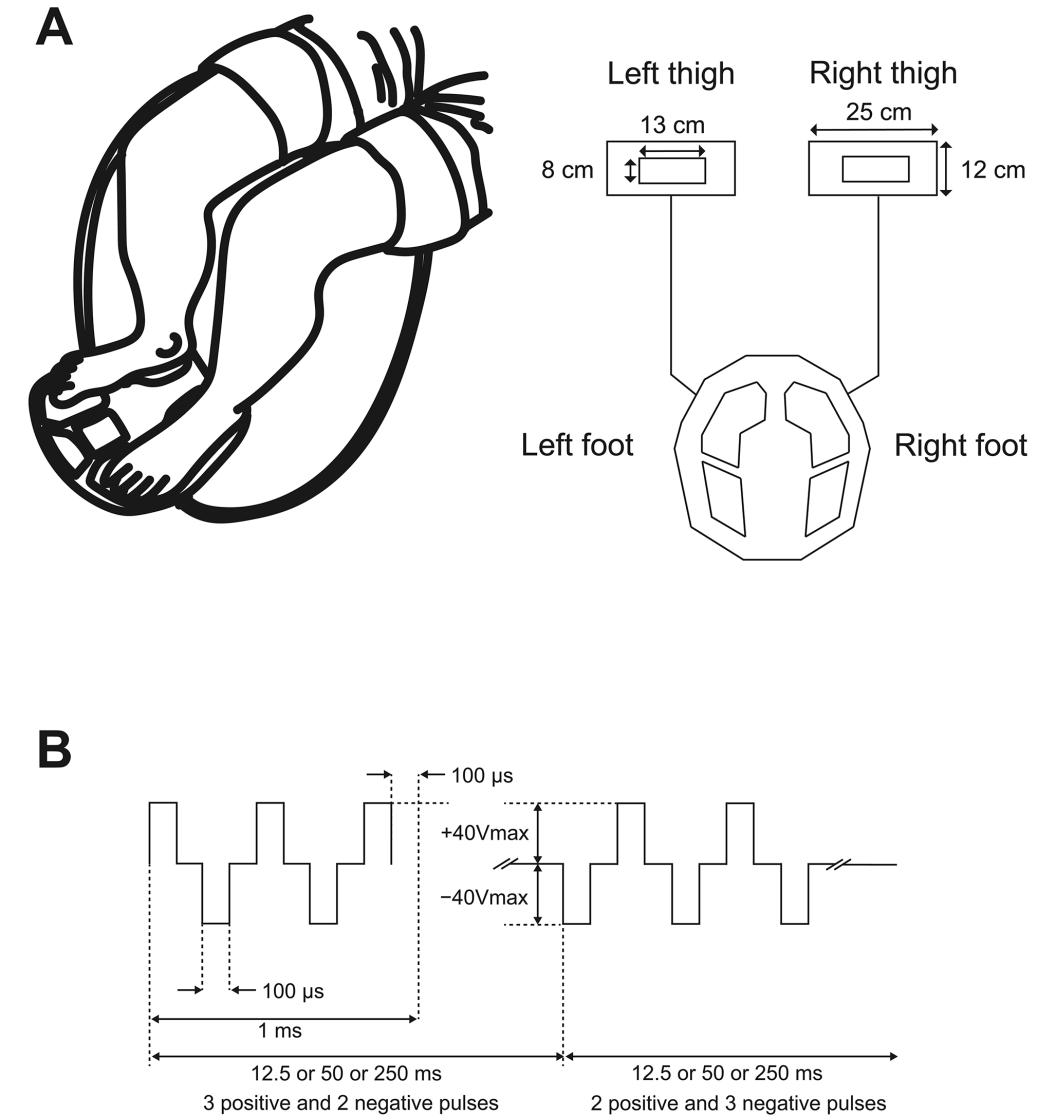
Position of electrodes and protocol of electrical muscle stimulation In the seated position, the electrodes were positioned at the midpoints of the bilateral femurs and the soles of the feet of the participants (**A**). The protocol of electrical muscle stimulation (**B**). The repetition time of pulse series varied between 12.5 ms (80 Hz), 50 ms (20 Hz), and 250 ms (4 Hz)

Serum BDNF and cathepsin-B levels were compared in all participants at four EMS frequencies: (1) 4 Hz, (2) 20 Hz, (3) 80 Hz, and (4) control condition (no stimulation). The EMS device used was a custom-made portable EMS device (MTG Ltd, Nagoya, Japan) for stimulation of the entire lower limbs. With participants in a seated position, the stimulation electrodes were positioned at the midpoints of the thighs and on the plantar surface of the feet (Fig. 1A). For each stimulation condition, 5 rectangular pulses were delivered with amplitudes of + 40 V and − 40 V and a pulse duration of 100 μs for 20 min. Positive and negative pulses alternated in these series. The stimulation duration varied between 250 ms (4 Hz condition), 50 ms (20 Hz condition), and 12.5 ms (80 Hz condition) (Fig. 1B). In this study, only the frequency changed between sessions, and a unified pulse duration of 100 μs was used in all conditions. Plantar flexion of the ankles and contraction of the quadriceps muscles were observed during stimulation of the entire lower limb. Sessions were separated by at least one week to avoid crossover effects. The control condition consisted of 20 min of quiet sitting. The interventions were performed in a randomized order. Participants were asked if they experienced any pain or discomfort during each session.

**Fig. 2:**
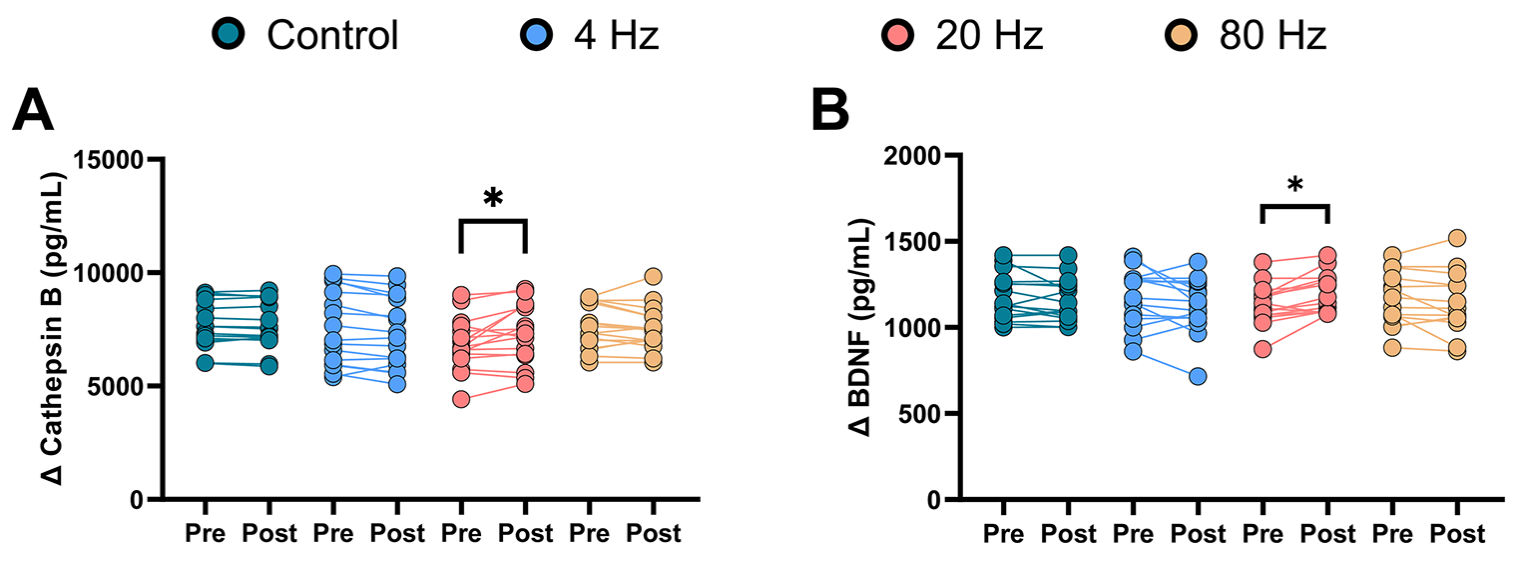
Comparison of pre- and postintervention cathepsin-B and BDNF levels between conditions The 20 Hz intervention resulted in significantly higher cathepsin-B (**A**) and BDNF (**B**) levels at postintervention than at preintervention. However, the other interventions and the control did not result in significant pre-post differences * *p* < 0.05, ** *p* < 0.01

**Fig. 3:**
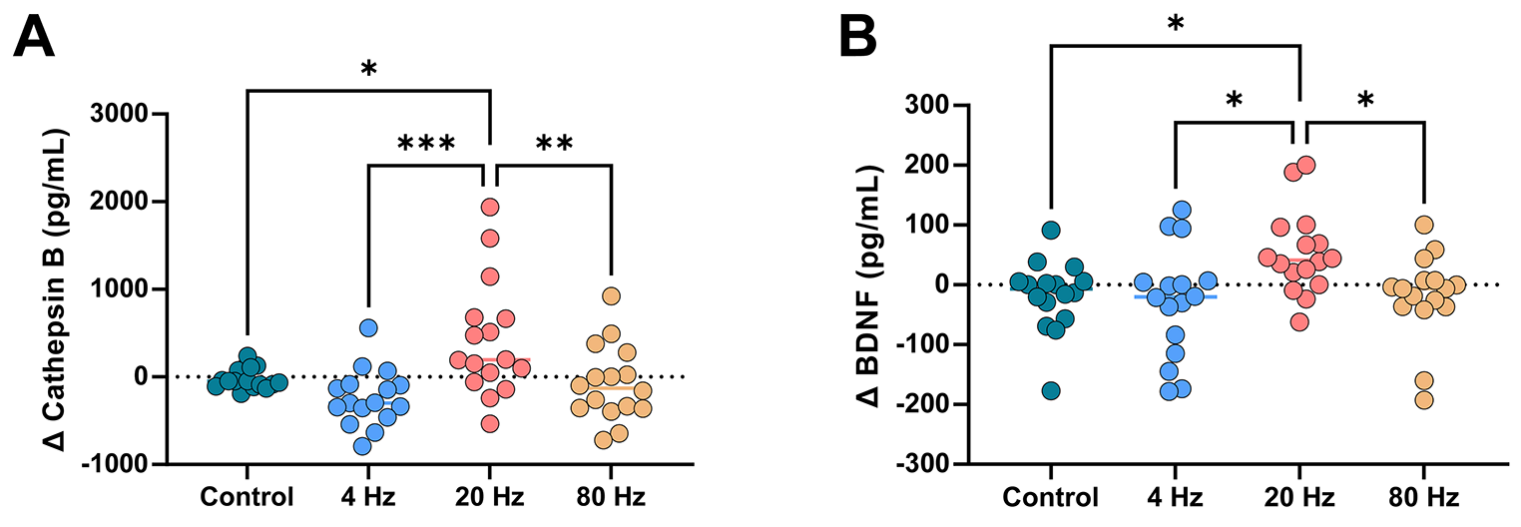
Comparison of changes in cathepsin-B and BDNF levels among groups The 20 Hz condition resulted in significantly higher changes in cathepsin-B (**A**) and BDNF (**B**) levels than the other interventions and the control condition * *p* < 0.05, ** *p* < 0.01, *** *p* < 0.001

### Measures of serum BDNF and cathepsin-B

Pre- and post-test blood samples for analysis were collected between 15:00 and 16:00 p.m., and participants were instructed to avoid excessive exercise and caffeine intake prior to blood collection. Blood samples were collected from the median cubital vein into vacuum tubes for measurement of BDNF and cathepsin-B levels. Blood samples were collected before and immediately after EMS interventions. Blood samples were also collected before and after 20 min of sitting quietly in the control condition. After blood collection, the blood samples were allowed to clot at room temperature for 30 min, and the serum was separated by centrifuging the vacuum tubes at 2,000 rpm. The serum samples were analyzed using enzyme-linked immunosorbent assays (ELISAs) according to the manufacturer’s instructions (Human BDNF ELISA kit (ab212166) and Human Cathepsin-B SLISA kit (ab272205), Abcam Inc., Cambridge, UK). ∆ cathepsin-B and ∆ BDNF were calculated as the difference the between post- and preintervention levels.

### Statistical analysis

All statistical analyses were conducted with GraphPad Prism version 9 (GraphPad Software Inc, California, USA). We used a two-way (intervention (4 H, 20 Hz, 80 Hz, and control) $\times$ period (pre and post) repeated-measures analysis of variance (ANOVA) to analyze the serum BDNF and cathepsin-B. The differences between each factor were analyzed by the Bonferroni post hoc test. The between-group differences in ∆ cathepsin-B and ∆ BDNF were examined using one-way ANOVA. For multiple comparisons, one-way ANOVA with a Tukey–Kramer test was used. The significance criterion was *p* < 0.05.

## Results

Participants did not experience any adverse events (e.g., muscle pain) as a result of the EMS interventions.

There was a significant interaction between the intervention $\times$ period of cathepsin B (*F* = 6.760, *p* < 0.001, *η*^*2*^ = 0.253) and BDNF (*F* = 5.071, *p* = 0.0034, *η*^*2*^ = 0.202). The 20 Hz condition resulted in significantly higher cathepsin B and BDNF levels posttreatment compared to pretreatment (*p* = 0.001, 95% CI = − 695.9 to − 142.0 pg/ml and *p* = 0.016, 95% CI = − 108.6 to − 8.356 pg/ml, respectively, Fig 2A and B). Conversely, the control and other conditions did not result in significant pre-post differences (Cathepsin B: control, *p* = 0.999, 95% CI = − 256.9 to 296.9 pg/ml; 4 Hz, *p* = 0.124, 95% CI = − 40.92 to 513.0 pg/ml; 80 Hz, *p* = 0.922, 95% CI = − 198.9 to 355.0 pg/ml; BDNF: control, *p* = 0.836, 95% CI = − 32.23 to 68.00 pg/ml; 4 Hz, *p* = 0.130, 95% CI = − 7.849 to 92.37 pg/ml; 80 Hz, *p* = 0.788, 95% CI = − 30.58 to 69.64 pg/ml).

A one-way ANOVA was used to compare levels between the groups, focusing on the degree of change in each condition. The 20 Hz condition produced significantly higher cathepsin-B levels than the control (*p* = 0.028, 95% CI = − 8.42.0 to − 35.88 pg/ml), 4 Hz (*p* < 0.001, 95% CI = − 1058.0 to 251.9 pg/ml), and 80 Hz (*p* = 0.01, 95% CI = 93.9 to 900.0 pg/ml) conditions (Fig. 3A). Furthermore, the 20 Hz condition produced significantly higher BDNF levels than the control (*p* = 0.046, 95% CI = − 139.3 to − 0.898 pg/ml), 4 Hz (*p* = 0.014, 95% CI = − 151.2 to − 12.78 pg/ml), and 80 Hz (*p* = 0.039, 95% CI = 2.541 to 140.9 pg/ml) conditions (Fig. 3B).

## Discussion

This study demonstrated the influence of the stimulation frequency of EMS on serum cathepsin-B and BDNF levels in healthy young adults. The novel result of this study was that stimulation at 20 Hz was effective in increasing serum cathepsin-B and BDNF levels. This finding partially supports our hypothesis. Interestingly, the increase in stimulation at 80 Hz was relatively low, and myokine production was not frequency dependent.

We found that stimulation at 20 Hz resulted in significantly higher production of serum cathepsin-B and BDNF. This finding has important implications for the effectiveness of 20 Hz for myokine production. Previous studies have reported that EMS at 4 Hz and 20 Hz increased serum BDNF levels (Miyamoto et al. 2018a; Nishikawa et al. 2021b, 2023). However, there have been no reports comparing myokine production at different EMS frequencies. Furthermore, to our knowledge, there have been no reports investigating EMS-induced changes in cathepsin-B production. Myokines such as BDNF and cathepsin-B have been reported to improve memory in several previous studies (Moon et al. 2016; Loprinzi and Frith 2019; Cefis et al. 2019; Okamoto et al. 2021). Numerous benefits of BDNF, including nerve regeneration and increased muscle protein synthesis, have been reported (Pedersen and Fischer 2007; Guo et al. 2021). Furthermore, BDNF has been implicated in mediating exercise effects in the hippocampus and is an important factor in memory enhancement (Loprinzi and Frith 2019). Recently, it has been reported that running induces cathepsin-B production in skeletal muscle, which affects memory (Moon et al. 2016). Cathepsin-B has also been shown to penetrate the blood–brain barrier and promote BDNF production in the brain, and a positive correlation was observed between the rate of improvement in cognitive function before and after exercise and the degree of increase in cathepsin-B levels in a human study (Hasegawa et al. 2011; Nishikawa et al. 2021a). These findings indicate that cathepsin-B production is an important factor in improving cognitive function; therefore, the increase in cathepsin-B levels observed after EMS is a very important finding. EMS has been widely used in the rehabilitation and sports fields for muscle strengthening, and in recent years, intervention studies have been conducted in the nervous system from the perspective of the effect of EMS on myokine levels (Nishikawa et al. 2019, 2021a, 2023). The finding that EMS contributes to myokine production is consistent with these previous studies.

In this study, the degree of change in BDNF levels was modest at best, although it was statistically significant. On the other hand, we identified a large degree of change in cathepsin-B levels. A previous study reported that cathepsin-B penetrates the blood‒brain barrier and promotes BDNF production in the brain (Moon et al. 2016). A correlation between BDNF levels in the brain and in the blood has also been reported, and capturing changes in BDNF levels may be more effective if the levels are measured after a waiting period rather than immediately after an EMS intervention. In our previous study, we observed that BDNF levels were highest 20 min after the end of EMS intervention (Nishikawa et al. 2023). These findings suggest that there is a temporal difference in the expression levels of BDNF and cathepsin-B.

Frequency is one of the parameters with a substantial impact on the efficacy of EMS (Moritani et al. 1985; Doucet et al. 2012); in clinical practice, EMS is usually performed using a range of frequencies between 20 and 50 Hz (Baker et al. 1988; de Kroon et al. 2005). Previous studies have reported that low-frequency EMS (20 Hz) has the potential to elicit more effective muscular improvement than high-frequency EMS (50–80 Hz) (Rebai et al. 2002; Hasegawa et al. 2011). Based on these findings, EMS administered for muscle-strengthening effects often utilizes a low frequency (20 Hz) (Hasegawa et al. 2011; Nishikawa et al. 2021a). However, there have been no previous studies examining the effective frequencies for myokine production. This study is the first report to focus on myokine production and to examine the effective EMS frequency. The results of this study indicate that 20 Hz contributes the most to myokine production, while 4 Hz and 80 Hz have a less immediate effect. High-frequency EMS has been reported to produce muscle fatigue in a short period of time (20–30 s), making it impossible to maintain muscle torque for extended periods of time (Moritani et al. 1985; Bigland-Ritchie et al. 2000). This finding suggests that in longer-duration EMS interventions (e.g., 20 min), high-frequency stimulation is not able to induce sustained effective muscle contractions due to the high strain on the muscles. On the other hand, for low-frequency EMS (4 Hz), the difference in the number of stimulated muscle fibers may be due to the difference in muscle contraction patterns (i.e., fused and/or unfused tetanus) induced by different stimulation frequencies. EMS-induced muscle contractions can be divided into fused and/or unfused tetanic contractions (Basford 2001). Type II fibers have been reported to have a higher frequency of fused tetanus than type I fibers, and it has been suggested that stimulation of type II fibers with lower-frequency EMS (4 Hz) is difficult (Orizio et al. 1989; Kossev et al. 1994; Yoshitake and Moritani 1999). Watanabe et al. reported that stimulation at approximately 14.2 Hz is required to produce fused tetanus in the soleus muscle (Watanabe et al. 2017). These findings suggested that the frequency does not have to be high or low to properly stimulate muscle fibers but that there is an appropriate frequency. Based on the results of this study, 20 Hz may be an indicator of adequate stimulation of muscle fibers.

This study has several limitations. First, this study recruited only young adults. Muscle composition is known to change with aging (Lexell and Taylor 1991; Bougea et al. 2016), and myokine production also decreases with aging (Shimada et al. 2014). Therefore, the effectiveness of EMS may differ in elderly people. Another limitation of this study is that it is unclear whether the induced increases in myokine levels will lead to long-term improvements in neurological function. Future studies should include long-term intervention trials in a wide range of age groups to determine the effects on cognitive function. Finally, 300 myokines that may be physiologically relevant, such as interleukin 6, myostatin, growth differentiation factor 11, etc., have been identified. We have focused only on BDNF and cathepsin-B. It remains to be seen whether other myokines will exhibit similar results.

## Conclusion

Our study investigated how the frequency of EMS affects the level of myokines in the blood of young adults. We found that EMS at 20 Hz is effective at increasing myokine levels. Myokines have been reported to contribute to muscle protein synthesis, nerve regeneration and protection (Severinsen and Pedersen 2020) and improvement of cognitive function (Wang and Holsinger 2018). Future studies will explore the effects of this intervention in patient populations.
